# Tea intake shows no genetic causal effect on stroke: A two-sample Mendelian randomization study based on STROBE-MR guidelines

**DOI:** 10.1097/MD.0000000000044398

**Published:** 2025-09-05

**Authors:** Yang Liu, Xinhong Zhang, Yuehui Liu, Dongwei Zhang

**Affiliations:** a Second Department of Neurology, Affiliated Hospital of Inner Mongolia Minzu University, Tongliao, China.

**Keywords:** Mendelian randomization, prognosis, single-nucleotide polymorphism, stroke, tea intake

## Abstract

Observational studies have reported inconsistent links between tea intake and stroke risk. We applied two-sample Mendelian randomization (MR) to clarify whether the association is causal. Following STROBE-MR guidelines, we extracted genome-wide association study (GWAS) summary statistics for tea intake (UK Biobank, *n* = 447,485; GWAS ID ukb-b-6066) and stroke (UK Biobank, *n* = 462,933; GWAS ID ukb-b-6358), both of European ancestry. Thirty-six independent single-nucleotide polymorphisms (SNPs) meeting genome-wide significance (*P* < 5 × 10^−8^), linkage disequilibrium threshold *r*² < 0.001 (10,000 kb), and *F* > 10 were selected as instrumental variables (IVs). Causal estimates were calculated using inverse variance weighting (IVW) as the primary analysis and weighted median, MR-Egger, simple mode, and weighted mode as complementary methods. Heterogeneity (Cochran *Q* and Rücker *Q*), horizontal pleiotropy (MR-Egger intercept), and leave-one-out sensitivity analyses were performed to evaluate robustness. IVW indicated no causal effect of tea intake on stroke (odds ratio [OR] = 0.997, 95% confidence interval [CI]: 0.991–1.004, *P* = .381). Findings were consistent across MR-Egger (OR = 0.984, 95% CI: 0.953–1.015, *P* = .306), weighted median, simple mode, and weighted mode analyses. No significant heterogeneity was detected (Cochran *Q P* = .176; Rücker *Q P* = .181), and the MR-Egger intercept showed no evidence of horizontal pleiotropy (*P* = .386). Leave-one-out and funnel plot assessments confirmed result stability and minimal bias. Genetic evidence from this large, two-sample MR study does not support a causal relationship between habitual tea consumption and stroke risk. Public health strategies aimed at stroke prevention should therefore not rely on promoting tea intake alone.

## 1. Introduction

Currently, stroke has become a common and frequently occurring disease in China. Epidemiological surveys indicate that the incidence of stroke in China is 5 times that of coronary heart disease. Stroke is characterized by high disability, high incidence, and relatively high mortality.^[1]^ It not only consumes a substantial amount of medical resources but also imposes a significant economic burden. Therefore, primary prevention of stroke is a top priority in public health.^[2,3]^

Tea is one of the most widely consumed beverages globally, and its consumption is increasing annually.^[4]^ preclinical and in vivo studies indicate that bioactive catechins in tea – particularly epigallocatechin-3-gallate – can cross the blood–brain barrier and exert antioxidant, anti-inflammatory, and neuroprotective actions on central nervous system cells.^[5]^ As a major producer and exporter of tea, China has a large population of tea consumers. Given the widespread consumption of tea, it is crucial to understand its potential health impacts on different populations. Numerous studies have explored the relationship between tea consumption and the incidence of stroke, but the findings remain controversial. Some studies have produced contradictory results, and no consensus has been reached.^[6–11]^

## 2. Materials and methods

### 2.1. Study design

This study followed the STROBE-MR reporting guidelines, Mendelian randomization (MR) is increasingly used in clinical disease research, relying on the random inheritance of alleles to create fixed genetic distributions.^[12,13]^ This allows genetic variants to serve as instrumental variables (IVs), minimizing reverse causality and confounding factors. This study uses a two-sample MR approach to explore the causal link between tea consumption and stroke, aiming to inform future stroke prevention strategies.

### 2.2. Data source

The database used in this study is the IEU OPEN GWAS public database (https://gwas.mrcieu.ac.uk/), which includes the genome-wide association study (GWAS) data on tea consumption (GWAS ID: ukb-b-6066) and stroke (GWAS ID: ukb-b-6358). All analyses used publicly available, de-identified GWAS summary statistics; therefore IRB approval and informed consent were waived. Detailed information on these 2 datasets is provided in Table 1.

**Table 1:** Brief information on the two-sample MR study GWAS.

	GWAS ID	Consortium	Sample size	Number of SNPs	Population
Tea intake	ukb-b-6066	MRC-IEU	447,485	9,851,867	European
Stroke	ukb-b-6358	MRC-IEU	462,933	9,851,867	European

GWAS = genome-wide association study, MR = Mendelian randomization.

### 2.3. Instrumental-variable selection

Selection of instrumental variables: The selection of instrumental variables must adhere to the 3 core assumptions of Mendelian randomization, as follows: Assumption 1 – single-nucleotide polymorphisms (SNPs) are strongly associated with tea consumption; Assumption 2 – SNPs are not associated with confounding factors; Assumption 3 – SNPs affect stroke incidence only through tea consumption and are not related to other confounding factors. To meet the above 3 assumptions, the selection of SNPs must adhere to the genome-wide statistical threshold of<5 × 10^−8^. To prevent linkage disequilibrium, a linkage disequilibrium threshold of *r*^2^ < 0.001, and a distance of 10,000 kb. Additionally, to avoid the generation of weak instrumental variables, an *F*-statistic >10 is applied. The formula for calculating *F* is as follows: $F=\frac{N-K-1}{K}\times \frac{R^{2}}{1-R^{2}}$,where N is the sample data for tea consumption, *K* is the number of SNPs, and *R*^2^ is the proportion of variance in tea consumption explained by the SNPs. The formula for calculating *R*^2^ is as follows: *R*^2^ = 2(1 − EAF) = ×EAF×β^2^, where EAF is the effect allele frequency of the SNP, β is the effect size of the allele, and outlier detection is performed using MR-PRESSO to exclude outlier SNPs. Finally, the included SNPs are checked using ldtrait (https://ldlink.nih.gov/?tab=ldtrait) to ensure they do not violate assumptions (2) and (3).

### 2.4. Statistical analysis

Causal effects of genetically predicted tea intake on stroke were estimated with 5 two-sample Mendelian randomization (MR) methods: inverse variance weighting (IVW, primary), MR-Egger regression, weighted median, simple mode, and weighted mode. Results are reported as odds ratios (ORs) with 95% confidence intervals.

Between-instrument heterogeneity was assessed with Cochran *Q* (IVW framework) and Rücker *Q* (MR-Egger framework). Horizontal pleiotropy was evaluated with the MR-Egger intercept and the MR-PRESSO global test. Robustness was further examined by leave-one-out analysis, and potential small-study bias was visualized with funnel and single-SNP plots. All computations were performed in R version 4.3.1 (R Foundation for Statistical Computing, Tongliao, Inner Mongolia, China) using the two-sample MR (v0.5.6) and MR-PRESSO (v1.0) packages. A 2-sided *P* < .05 was considered statistically significant.

### 2.5. Sensitivity analyses

Sensitivity analyses were performed to evaluate the robustness of the causal estimates. Cochran *Q* and Rücker *Q* tests assessed heterogeneity across instruments. Horizontal pleiotropy was examined with the MR-Egger intercept test and MR-PRESSO global test. Leave-one-out analysis, funnel plots, and single-SNP plots were generated to detect influential variants and small-study effects.

### 2.6. Ethics statement

All analyses were performed with publicly available, de-identified genome-wide association study (GWAS) summary statistics (UK Biobank accession numbers ukb-b-6066 and ukb-b-6358). No new individual-level or identifiable human data were collected. Consequently, this secondary analysis was exempt from institutional review board (IRB) review and from the requirement for written informed consent, in accordance with the Declaration of Helsinki and local regulations.

## 3. Results

### 3.1. Instrumental-variable selection

Instrumental-variable selection results: After removing linkage disequilibrium and conducting *F*-statistic testing (with instrumental variables having *F* > 10), a total of 39 SNPs were initially included. These 39 SNPs were then subjected to outlier detection using MR-PRESSO, and outlier SNPs (rs9624470) with a *P*-value < 0.05 were excluded. Subsequently, the remaining SNPs were individually input into ldtrait (https://ldlink.nih.gov/?tab=ldtrait) to check whether they violated core assumptions (2) and (3). SNPs rs4410790 and rs2472297 were excluded, as they could affect stroke through BMI as an exposure pathway. Finally, 36 SNPs were retained (see Table 2 for details).

**Table 2:** Final included SNPs information.

SNPs	chr	EA	OA	EAF	Tea intake			Stroke			*F*
					β	SE	*P*	β	SE	*P*	
rs10741694	11	C	T	0.628	0.015	2.194 × 10^−3^	7.900 × 10^−12^	5.372 × 10^−5^	2.458 × 10^−4^	8.300 × 10^−1^	47.070
rs10752269	10	A	G	0.506	−0.013	2.120 × 10^−3^	1.300 × 10^−9^	−3.397 × 10^−4^	2.375 × 10^−4^	1.500 × 10^−1^	37.071
rs10764990	10	A	G	0.607	−0.012	2.169 × 10^−3^	1.900 × 10^−8^	2.447 × 10^−4^	2.430 × 10^−4^	3.100 × 10^−1^	31.723
rs11164870	1	G	C	0.605	−0.012	2.182 × 10^−3^	4.200 × 10^−8^	5.538 × 10^−4^	2.446 × 10^−4^	2.400 × 10^−2^	30.606
rs1156588	2	G	A	0.210	−0.015	2.603 × 10^−3^	2.900 × 10^−9^	−3.524 × 10^−4^	2.918 × 10^−4^	2.300 × 10^−1^	35.467
rs11587444	1	G	A	0.393	0.014	2.171 × 10^−3^	1.000 × 10^−10^	−1.479 × 10^−4^	2.434 × 10^−4^	5.400 × 10^−1^	42.059
rs12591786	15	T	C	0.159	−0.018	2.942 × 10^−3^	3.700 × 10^−10^	−1.222 × 10^−4^	3.295 × 10^−4^	7.100 × 10^−1^	40.656
rs13282783	8	T	C	0.286	−0.014	2.354 × 10^−3^	7.900 × 10^−9^	4.801 × 10^−4^	2.637 × 10^−4^	6.900 × 10^−2^	33.717
rs132904	22	C	G	0.779	0.017	2.553 × 10^−3^	7.800 × 10^−11^	−2.378 × 10^−4^	2.858 × 10^−4^	9.300 × 10^−1^	42.513
rs1453548	11	A	T	0.665	−0.013	2.250 × 10^−3^	3.000 × 10^−9^	−2.618 × 10^−4^	2.521 × 10^−4^	3.000 × 10^−1^	35.494
rs1481012	4	G	A	0.112	−0.026	3.356 × 10^−3^	5.300 × 10^−15^	1.340 × 10^−4^	3.761 × 10^−4^	7.200 × 10^−1^	61.412
rs17245213	11	A	G	0.208	−0.015	2.609 × 10^−3^	2.000 × 10^−8^	3.093 × 10^−4^	2.923 × 10^−4^	2.900 × 10^−1^	31.642
rs17576658	13	A	G	0.247	−0.013	2.457 × 10^−3^	4.100 × 10^−8^	−5.805 × 10^−4^	2.751 × 10^−4^	3.500 × 10^−2^	30.261
rs17685	7	A	G	0.278	0.023	2.362 × 10^−3^	1.600 × 10^−22^	−2.976 × 10^−5^	2.647 × 10^−4^	9.100 × 10^−1^	95.486
rs2117137	3	G	A	0.405	0.013	2.156 × 10^−3^	1.700 × 10^−9^	−2.408 × 10^−4^	2.416 × 10^−4^	3.200 × 10^−1^	36.426
rs2273447	20	T	A	0.204	0.017	2.634 × 10^−3^	3.300 × 10^−11^	−3.264 × 10^−4^	2.952 × 10^−4^	2.700 × 10^−1^	44.332
rs2279844	17	A	G	0.379	−0.012	2.183 × 10^−3^	4.000 × 10^−8^	5.943 × 10^−5^	2.445 × 10^−4^	8.100 × 10^−1^	30.284
rs2351187	10	A	G	0.319	0.013	2.282 × 10^−3^	1.600 × 10^−8^	−8.158 × 10^−5^	2.557 × 10^−4^	7.500 × 10^−1^	32.364
rs2478875	6	G	A	0.209	0.022	2.611 × 10^−3^	5.100 × 10^−17^	−6.996 × 10^−4^	2.927 × 10^−4^	1.700 × 10^−2^	70.875
rs2645929	13	G	A	0.813	−0.015	2.717 × 10^−3^	3.500 × 10^−8^	−2.024 × 10^−4^	3.045 × 10^−4^	5.100 × 10^−1^	30.544
rs2783129	13	G	C	0.485	−0.012	2.133 × 10^−3^	3.800 × 10^−8^	−2.298 × 10^−4^	2.389 × 10^−4^	3.400 × 10^−1^	30.776
rs34619	5	A	G	0.431	0.012	2.138 × 10^−3^	4.300 × 10^−8^	6.408 × 10^−5^	2.395 × 10^−4^	7.900 × 10^−1^	30.105
rs4808193	19	C	T	0.335	0.015	2.247 × 10^−3^	1.700 × 10^−11^	−6.484 × 10^−6^	2.517 × 10^−4^	9.800 × 10^−1^	45.576
rs4817505	21	C	T	0.390	0.015	2.175 × 10^−3^	4.200 × 10^−12^	2.253 × 10^−4^	2.436 × 10^−4^	3.600 × 10^−1^	48.345
rs56188862	1	C	T	0.387	−0.016	2.175 × 10^−3^	4.300 × 10^−13^	−9.729 × 10^−5^	2.437 × 10^−4^	6.900 × 10^−1^	52.742
rs56348300	9	G	C	0.185	0.016	2.732 × 10^−3^	6.100 × 10^−9^	1.091 × 10^−4^	3.061 × 10^−4^	7.200 × 10^−1^	33.987
rs57462170	3	A	G	0.109	0.019	3.406 × 10^−3^	1.900 × 10^−8^	−4.536 × 10^−4^	3.816 × 10^−4^	2.300 × 10^−1^	31.821
rs57631352	19	G	A	0.297	−0.013	2.321 × 10^−3^	1.700 × 10^−8^	3.650 × 10^−5^	2.600 × 10^−4^	8.900 × 10^−1^	32.078
rs6829	13	T	C	0.596	−0.012	2.165 × 10^−3^	3.700 × 10^−8^	1.225 × 10^−4^	2.426 × 10^−4^	6.100 × 10^−1^	30.598
rs713598	7	G	C	0.402	0.013	2.157 × 10^−3^	5.200 × 10^−10^	−3.180 × 10^−4^	2.418 × 10^−4^	1.900 × 10^−1^	38.625
rs72797284	5	G	A	0.271	−0.017	2.384 × 10^−3^	7.000 × 10^−13^	−1.094 × 10^−4^	2.671 × 10^−4^	6.800 × 10^−1^	51.771
rs7757102	6	G	A	0.555	−0.012	2.133 × 10^−3^	3.100 × 10^−8^	^−8^.515 × 10^−6^	2.391 × 10^−4^	9.700 × 10^−1^	30.794
rs9302428	16	G	C	0.636	0.012	2.201 × 10^−3^	2.600 × 10^−8^	6.725 × 10^−4^	2.466 × 10^−4^	6.400 × 10^−3^	31.079
rs9648476	7	A	G	0.623	0.013	2.185 × 10^−3^	1.100 × 10^−8^	2.618 × 10^−6^	2.449 × 10^−4^	9.900 × 10^−1^	32.855
rs977474	12	T	C	0.834	0.022	2.856 × 10^−3^	2.400 × 10^−14^	−2.093 × 10^−4^	3.199 × 10^−4^	5.100 × 10^−1^	58.863
rs9937354	16	A	G	0.424	−0.014	2.143 × 10^−3^	4.900 × 10^−11^	2.092 × 10^−4^	2.401 × 10^−4^	3.800 × 10^−1^	43.413

Chr = chromosome, EA = effect allele, EAF = effect allele frequency, OA = other allele, SE, standard error, SNP = single-nucleotide polymorphism.

### 3.2. Causal effect estimates

**Figure 1.**
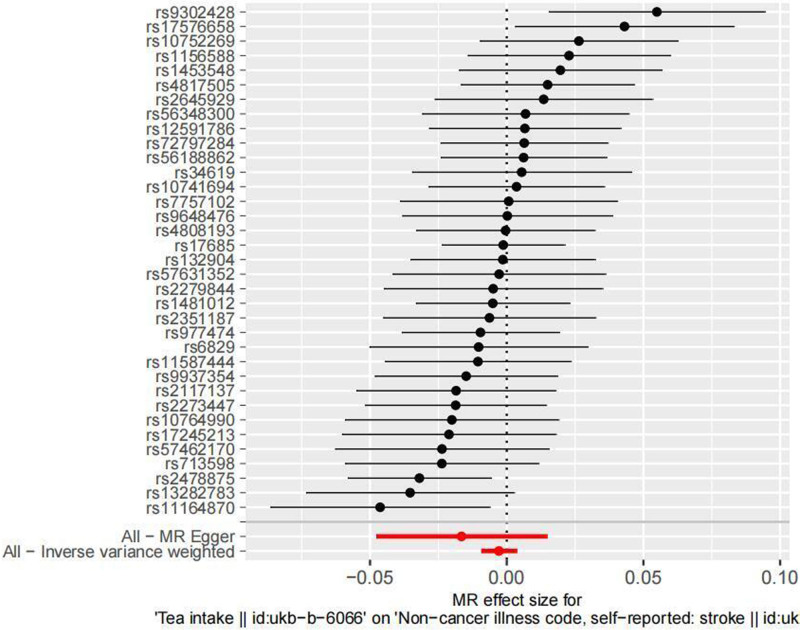
Forest map of two-sample MR results. MR = Mendelian randomization.

**Figure 2.**
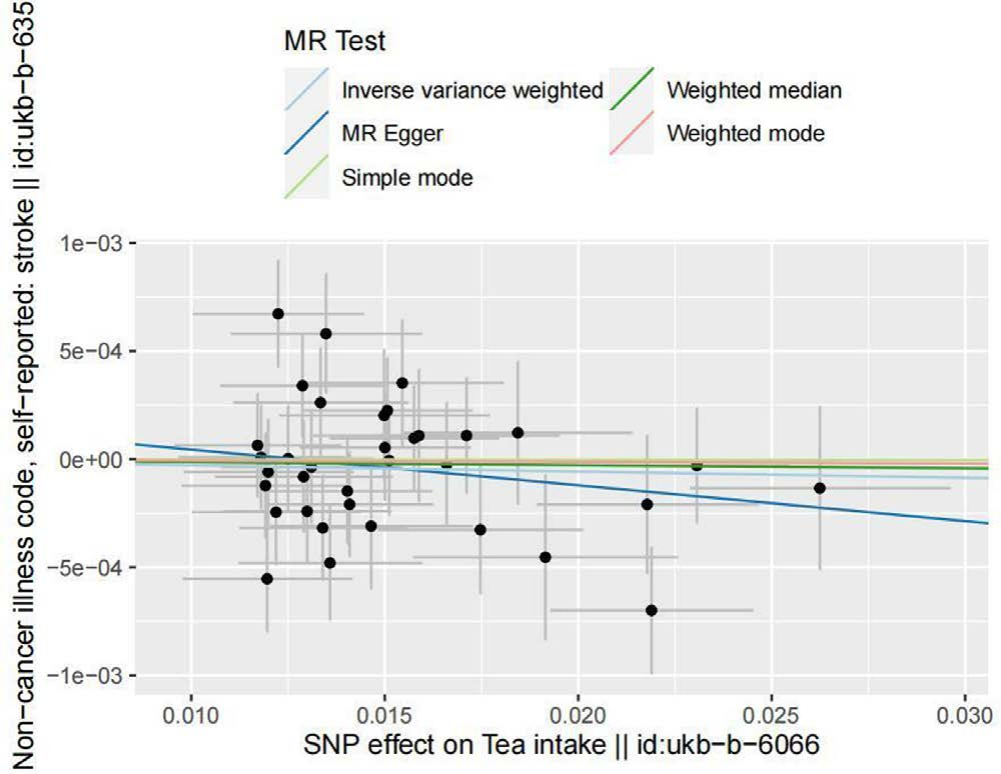
Scatter plot of MR results for 2 samples. MR = Mendelian randomization.

MR analysis results: In determining the causal relationship, the results are as follows: IVW (OR = 0.997, 95% CI = 0.991–1.004, *P* = .381), MR-Egger (OR = 0.984, 95% CI = 0.953–1.015, *P* = .306), weighted median (OR = 0.998, 95% CI = 0.990–1.007, *P* = .740), simple mode (OR = 0.999, 95% CI = 0.983–1.016, *P* = .989), weighted mode (OR = 0.999, 95% CI = 0.984–1.015, *P* = .935). The corresponding forest plot is shown in Figure 1. The scatter plot trends for the 5 MR analysis methods are consistent (see Fig. 2). These results indicate that there is no causal relationship between tea consumption and stroke incidence.

### 3.3. Heterogeneity and pleiotropy tests

Heterogeneity test: In the heterogeneity analysis, the Cochran *Q* test showed a *P*-value of .176 (>0.05), and the Rücker *Q* test showed a *P*-value of .181 (>0.05), indicating no significant heterogeneity. Reliability analysis: Pleiotropy analysis: The Egger intercept was used for pleiotropy testing, with the MR-Egger intercept being 0.00023, which is close to 0. The *P*-value was .386 (>0.05), indicating no evidence of pleiotropy.

### 3.4. Sensitivity analyses

**Figure 3.**
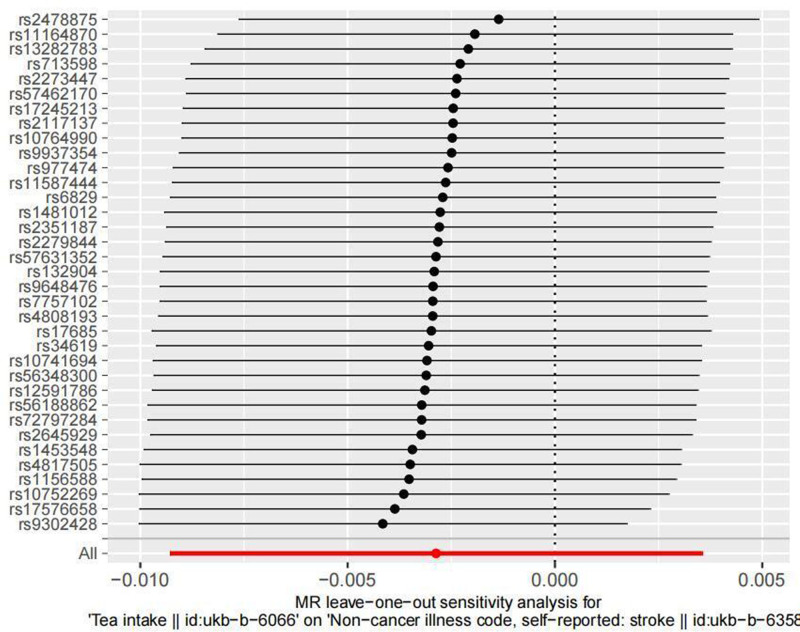
Excluding sensitivity analysis results one by one using the “leave-one-out”.

Sensitivity analysis: The leave-one-out method was applied by systematically excluding each SNP, and no significant impact on the results was observed. The detailed results are shown in Figure 3.

**Figure 4.**
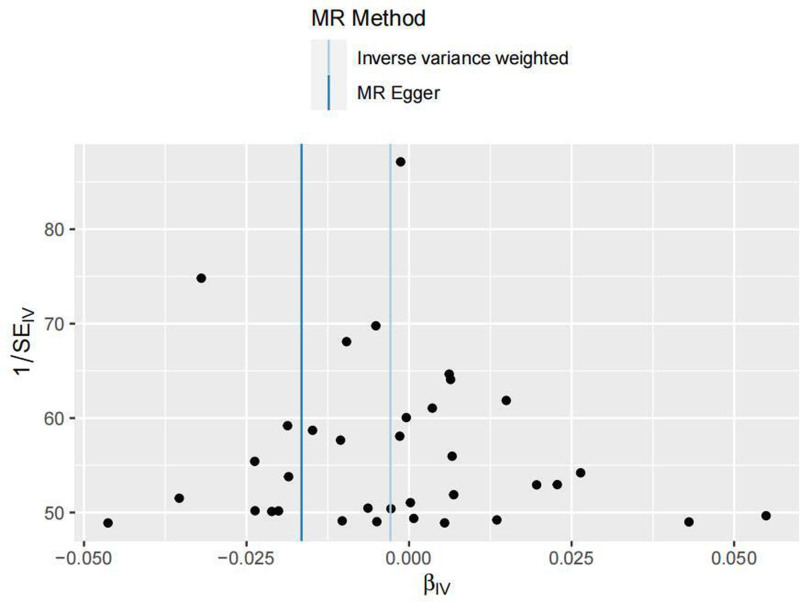
Funnel plot of MR results for 2 samples. MR = Mendelian randomization.

Additionally, the funnel plot (Fig. 4) shows that the effect sizes representing the causal relationship are symmetrically distributed, suggesting that the results are less likely to be influenced by potential bias.

## 4. Discussion

Brain tissue is highly vulnerable to damage from hypoxia and ischemia, with permanent injury occurring within 5 to 6 seconds.^[14–16]^ Stroke progresses rapidly, often leading to poor prognosis and high disability, which burdens both families and the healthcare system. Effective primary prevention is crucial. Stroke risk factors include age, family history, hypertension, smoking, diabetes, and poor diet, with diet being a key trigger.^[17–19]^

Tea is one of the most popular natural beverages in the world, with a wide variety of types and fermentation processes, including non-fermented green tea, semi-fermented oolong tea, and fully fermented black tea. Green tea currently accounts for 20% of the global tea consumption market, with major consumption in Asian countries, including South Korea, China, and Japan. Studies have been conducted in Asia on the relationship between green tea consumption and stroke risk. A joint cohort study in Japan, a case-control study in South Korea, and a cross-sectional study in China all found a negative correlation between green tea consumption and stroke risk.^[20–22]^ Additionally, some epidemiological studies have reported that green tea has beneficial effects on cardiovascular diseases, chemical-related diseases, blood pressure, lung cancer, and colorectal cancer.^[23]^ In studies suggesting that tea consumption can reduce the risk of stroke, the main mechanisms proposed include: tea may prevent atherosclerosis by protecting the arterial walls from the effects of high concentrations of low-density lipoprotein plasma, inhibiting monocyte migration to atherosclerotic lesion sites, suppressing smooth muscle cell proliferation, and improving endothelial function. However, the relationship between tea consumption and stroke risk remains inconsistent in large-scale observational studies in Europe.^[24]^ In the Netherlands EPIC_NL cohort of 37,514 individuals, a high tea consumption was not associated with stroke risk. However, in another cohort of 74,961 Swedish adults, high tea consumption (4 cups/d) was significantly associated with a reduced risk of total stroke.^[25]^ Whether tea consumption is associated with the occurrence of stroke remains inconclusive.

In this two-sample MR study, it was shown that tea consumption is not causally related to stroke incidence. The research team employed 5 MR analysis methods, and the trends in the scatter plots from all 5 methods were consistent. All 5 MR methods indicated no causal relationship between tea consumption and stroke incidence. To validate the reliability of the results, we conducted heterogeneity tests. In the heterogeneity analysis, the Cochran *Q* test yielded a *P*-value of .176 (>0.05), and the Rücker *Q* test showed a *P*-value of .181 (>0.05), indicating no significant heterogeneity. Sensitivity analysis was also performed, and to further assess whether there was substantial potential bias in this MR study, funnel plots were drawn. In the sensitivity analysis, the leave-one-out method was applied by systematically excluding each SNP, and no SNPs were found to significantly affect the results. Additionally, the funnel plot showed that the effect sizes representing the causal relationship were symmetrically distributed, suggesting a low likelihood of being influenced by potential bias. These results collectively indicate that the MR findings of this study are reliable, stable, and minimally affected by potential bias.

Regarding the conflicting findings between tea consumption and stroke incidence in traditional clinical studies, the author primarily attributes this to recall bias and reverse causality. This is because symptoms prior to a stroke diagnosis may alter tea-drinking habits. For traditional case-control studies, cohort studies, and even randomized controlled trials, it is inevitable that confounding factors and reverse causality influence the results. Previous observational studies reporting an association between tea consumption and stroke risk may have been affected by bias or confounding factors inherent in these studies. Some meta-analyses suggesting that tea consumption may influence the occurrence of stroke could be due to overlooking several inconsistent cohort studies and failing to comprehensively include all relevant studies in their analysis.^[26]^

The innovation of this study lies in analyzing the causal relationship between tea consumption and stroke incidence from the perspective of genetic epidemiology using a large sample size GWAS database. The conclusion drawn is that there is no causal relationship between tea consumption and stroke incidence. This helps prevent populations from blindly increasing tea consumption for stroke prevention based on potentially biased conclusions from observational studies, thereby contributing to the scientific prevention and treatment of stroke.

The limitations of this study include the fact that the GWAS data used are based on a European population, which makes it difficult to generalize the findings to other populations.

## 5. Conclusion

This study utilized a two-sample Mendelian randomization approach to investigate the causal relationship between tea intake and stroke incidence. Based on comprehensive genetic data and robust statistical analyses, the findings indicate no causal relationship between tea consumption and the risk of stroke. These results underscore the limitations of observational studies that often report associations influenced by confounding factors or reverse causality. By leveraging genetic epidemiology, this research contributes to a more accurate understanding of the health implications of tea consumption.

The findings provide valuable insights for public health initiatives, emphasizing that promoting tea consumption as a primary prevention strategy for stroke may lack scientific justification. However, the study’s applicability is constrained by its reliance on GWAS data from European populations, highlighting the need for further research across diverse ethnic groups.

## Acknowledgments

We thank the MiBioGen consortium and the IEU OPEN GWAS project for granting access to the publicly available summary statistics that made this work possible.

## Author contributions

**Conceptualization:** Xinhong Zhang.

**Data curation:** Yang Liu.

**Formal analysis:** Yang Liu.

**Funding acquisition:** Yang Liu.

**Investigation:** Yang Liu.

**Methodology:** Yang Liu.

**Project administration:** Yang Liu, Dongwei Zhang.

**Resources:** Dongwei Zhang.

**Supervision:** Xinhong Zhang, Yuehui Liu.

**Validation:** Xinhong Zhang, Yuehui Liu, Dongwei Zhang.

**Visualization:** Xinhong Zhang, Yuehui Liu, Dongwei Zhang.

**Writing – original draft:** Dongwei Zhang.

**Writing – review & editing:** Dongwei Zhang.

## References

[1] Ağaçkiran I Özdamar Y Demir MC. Role of transcranial Doppler ultrasonography in detecting and excluding ischemic stroke in the emergency department: a prospective observational study. Noro Psikiyatr Ars. 2024;62:119–24. 40583950 10.29399/npa.28723 PMC12205384

[2] Chao BH Yan F Hua Y. Stroke prevention and control system in China: CSPPC-Stroke program. Int J Stroke. 2021;16:265–72. 32223541 10.1177/1747493020913557

[3] Hu S Cui B Mlynash M Zhang X Mehta KM Lansberg MG. Stroke epidemiology and stroke policies in China from 1980 to 2017: a systematic review and meta-analysis. Int J Stroke. 2020;15:18–28. 31543073 10.1177/1747493019873562

[4] Pan SY Nie Q Tai HC. Tea and tea drinking: China’s outstanding contributions to mankind. Chin Med. 2022;17:1–40. 35193642 10.1186/s13020-022-00571-1 PMC8861626

[5] Musial C Kuban-Jankowska A Gorska-Ponikowska M. Beneficial properties of green tea catechins. Int J Mol Sci. 2020;21:1744. 32143309 10.3390/ijms21051744 PMC7084675

[6] Larsson SC Männistö S Virtanen MJ Kontto J Albanes D Virtamo J. Coffee and tea consumption and risk of stroke subtypes in male smokers. Stroke. 2008;39:1681–7. 18369170 10.1161/STROKEAHA.107.504183

[7] Tanabe N Suzuki H Aizawa Y Seki N. Consumption of green and roasted teas and the risk of stroke incidence: results from the Tokamachi–Nakasato cohort study in Japan. Int J Epidemiol. 2008;37:1030–40. 18832387 10.1093/ije/dyn211

[8] Liang W Lee AH Binns CW Huang R Hu D Zhou Q. Tea consumption and ischemic stroke risk: a case-control study in southern China. Stroke. 2009;40:2480–5. 19478218 10.1161/STROKEAHA.109.548586

[9] de Koning Gans JM Uiterwaal CSPM van der Schouw YT. Tea and coffee consumption and cardiovascular morbidity and mortality. Arterioscler Thromb Vasc Biol. 2010;30:1665–71. 20562351 10.1161/ATVBAHA.109.201939

[10] Kokubo Y Iso H Saito I. Impact of green tea and coffee consumption on reduced risk of stroke incidence in a Japanese population: the Japan Public Health Center-based cohort. Stroke. 2013;44:1369–74. 23493733 10.1161/STROKEAHA.111.677500

[11] Tian T Lv J Jin G; China Kadoorie Biobank Collaborative Group. Tea consumption and risk of stroke in Chinese adults: a prospective cohort of half a million men and women. Am J Clin Nutr. 2020;111:197–206. 31711152 10.1093/ajcn/nqz274 PMC7223259

[12] Richmond RC Smith GD. Mendelian randomization: concepts and scope. Cold Spring Harb Perspect Med. 2022;12:a040501. 34426474 10.1101/cshperspect.a040501 PMC8725623

[13] Brumpton B Sanderson E Heilbron K; Within-family Consortium. Avoiding dynastic, assortative mating, and population stratification biases in Mendelian randomization through within-family analyses. Nat Commun. 2020;11:3519. 32665587 10.1038/s41467-020-17117-4 PMC7360778

[14] Yamashiro K Kurita N Urabe T Hattori N. Role of the gut microbiota in stroke pathogenesis and potential therapeutic implications. Ann Nutr Metab. 2021;77:36–44. 34107468 10.1159/000516398 PMC8619778

[15] Ge Y Chen W Axerio-Cilies P Wang YT. NMDARs in cell survival and death: implications in stroke pathogenesis and treatment. Trends Mol Med. 2020;26:533–51. 32470382 10.1016/j.molmed.2020.03.001

[16] Chidambaram SB Rathipriya AG Mahalakshmi AM. Influence of gut dysbiosis in the pathogenesis and management of ischemic stroke. Cells. 2022;11:1239. 35406804 10.3390/cells11071239 PMC8997586

[17] Zhen X Zhao M Kawashima T. Effectiveness and safety of adjuvant therapy with t-PA in acute ischemic stroke: a systematic review and network meta-analysis. J Clin Question. 2024;1:89–97.

[18] Shi Q Cheng Q Chen C. Role of autophagy in the pathogenesis of ischemic stroke. Curr Neuropharmacol. 2021;19:629–40. 32727333 10.2174/1570159X18666200729101913 PMC8573746

[19] Barakli S Yön MI Ercan K. Relationship between collateral status, infarct growth and outcome in MCA occlusion assessed by CT angiography and perfusion imaging. Noro Psikiyatr Ars. 2024;62:41–7. 40046199 10.29399/npa.28643 PMC11877382

[20] Choi YH Laaker C Hsu M Cismaru P Sandor M Fabry Z. Molecular mechanisms of neuro-immune crosstalk in the pathogenesis of stroke. Int J Mol Sci . 2021;22:9486. 34502395 10.3390/ijms22179486 PMC8431165

[21] Lee J Kim Y. Association between green tea consumption and risk of stroke in middle-aged and older Korean men: the HEXA study. Prev Nutr Food Sci. 2019;24:24–31. 31008093 10.3746/pnf.2019.24.1.24 PMC6456242

[22] Lee SM Choi NK Yoon BW Park J-M Han M-K Park B-J. Impact of green tea consumption on prevention of hemorrhagic stroke. Neuroepidemiology. 2015;44:215–20. 26021303 10.1159/000381267

[23] Chen Z Li Y Zhao LC. Study on the association between tea consumption and stroke. Zhonghua Liu Xing Bing Xue Za Zhi. 2004;25:666–70. 15555387

[24] Shin S Lee JE Loftfield E. Coffee and tea consumption and mortality from all causes, cardiovascular disease and cancer: pooled analysis from the Asia Cohort Consortium. Int J Epidemiol. 2022;51:626–40. 34468722 10.1093/ije/dyab161 PMC9308394

[25] Pinheiro LC Reshetnyak E Akinyemiju T Phillips E Safford MM. Social determinants of health and cancer mortality in the REGARDS cohort. Cancer. 2022;128:122–30. 34478162 10.1002/cncr.33894 PMC9301452

[26] Larsson SC Virtamo J Wolk A. Black tea consumption and risk of stroke in women and men. Ann Epidemiol. 2013;23:157–60. 23295000 10.1016/j.annepidem.2012.12.006

